# Physical activity in paid work time for desk-based employees: a qualitative study of employers’ and employees’ perspectives

**DOI:** 10.1186/s12889-020-08580-1

**Published:** 2020-04-06

**Authors:** Gemma C. Ryde, Patricia Atkinson, Martine Stead, Trish Gorely, Josie M. M. Evans

**Affiliations:** 1 Faculty of Health Sciences and Sport, University of Stirling, Stirling, FK9 4LA Scotland, UK; 2 Department of Nursing, University of the Highlands and Islands, Inverness, IV3 5SQ Scotland, UK

**Keywords:** Qualitative, Workplace, Physical activity, Paid work time

## Abstract

**Background:**

Poor physical and mental health of employees create significant problems in the workplace. Physical activity (PA) has been shown as an effective strategy for preventing and treating numerous physical and mental health issues as well as work performance outcomes. However, there are many barriers to taking part in PA (such as lack of time) with participation rates typically low. Providing PA in paid work time might be a way to overcome these issues, yet employers’ and employees’ opinions of this concept are unknown. The aim of this study was to explore employee and employer perspectives of PA in paid work time.

**Methods:**

Workplaces were recruited through existing contacts on the research team. Focus groups and interviews were conducted with employees and managers at one University and two executive non-departmental public bodies in central Scotland with mainly desk-based employees. Both managers and employees were involved to gain perspectives throughout the organisational hierarchy and were interviewed separately to reduce social desirability bias. All discussions were digitally recorded and transcribed verbatim. Data were analysed thematically for both managers and employees but due to significant overlap in themes between the groups, these are reported together in the results.

**Results:**

Three out of five organisations approached took part in this qualitative study. Two individual interviews were held with strategic managers, five focus groups with middle managers (*n* = 16) and nine with employees (*n* = 45). Benefits were anticipated by managers and employees for both employees themselves and the organisation and included improved mental health, productivity and more favourable perceptions of the employer. Despite these widely acknowledged benefits, significant barriers were identified and included the structure and nature of the working day (high workload, front line job requirements), workplace culture and norms (resentment from colleagues, no break culture) and organisational concerns (cost of lost time, public perceptions).

**Conclusion:**

This study suggests that there are significant barriers to PA in paid work time. Whilst numerous anticipated benefits were conveyed by both employees and managers, PA in paid work time is unlikely to become common place until changes in attitudes and the culture towards movement at work occur.

## Background

Poor physical and mental health of employees create significant problems in the workplace. Work-related stress is the largest cause of sickness absence in the UK costing £13bn from lost days at work and productivity, with similarly high figures per population reported around the world including Australia, United States and across Europe [1, 2]. Musculoskeletal disorders also pose similar problems for workplaces in terms of productivity, with work-related musculoskeletal disorders accounting for 29% of all working days lost due to work-related ill health in the UK [3]. Many western workplace environments are predominantly desk-based with high levels of sitting and limited movement occurring during work hours [4–6]. This lack of physical activity (PA) and movement in desk-based workers is a concern given that workplace physical activity interventions can have positive effects on musculoskeletal pain [7, 8], depressive symptoms and anxiety [9], and work performance outcomes [10]. Increasing PA levels of desk-based employees could provide significant benefit to both workplaces and employees themselves and contribute towards improved population health.

However, review level evidence of intervention studies aiming to increase PA levels of employees in the workplace setting have shown only modest changes [11, 12]. These studies typically reported interventions where PA occurred within employees' discretionary time during working hours such as lunchtime. Several reasons for these modest changes have been suggested, with low participation rates in interventions a commonly reported theme. In a qualitative study by Kirwan et al. (2016) that surveyed employees’ attitudes toward regular PA in the workplace i.e. not part of a specific PA intervention, only 15% of employees reported participating in any form of PA during working hours [13]. Numerous studies have therefore aimed to investigate potential barriers to PA at work in order to increase participation. Consistent with national level PA surveillance data, lack of time is frequently cited as a significant barrier to participating in PA at work [13–16]. New insights into how to reduce this barrier and increase employee participation are therefore required.

Providing employees with time for PA not only during working hours but in *paid* work time might be a potential way to overcome the barrier of perceived lack of time and may improve participation rates. For the purpose of the current study, PA during working hours refers to existing discretionary time at work such as lunchtime. PA in paid work time includes time off in addition to existing breaks to specifically undertake physical activity. A systematic review of recruitment rates in workplace PA interventions suggested that interventions that provided employees with PA opportunities during paid work time had more favourable recruitment rate than those that did not (> 70% of employees recruited as a percentage of those in the workplace) [17]. One such study included in this review examined the effects of supervised group aerobics and strength training sessions twice per week for 9 months in paid work time on employees physical health [18]. The study findings showed positive outcomes in relation to body fat, dynamic muscle performance and cardio respiratory fitness, in addition to 100% of the workforce taking part in the study. Other studies have tried to incorporate PA activities into routine work practices. Yancey et al. 2004 implemented 10 min exercise breaks into meetings lasting more than 60 min [19]. They reported that 90% of employee who could take part did engage in the exercise session.

While there are examples of interventions that have adopted the approach of incorporating PA into paid work time with positive effects on participation rates and health outcomes, how this approach might work in the UK is unclear. Most of the research to date has taken place in the US and Scandinavia [18–21]. How or whether similar interventions could be translated into other countries such as the UK with potentially different work environments and cultures is unknown [22]. For example, in Denmark, offering employees sit-stand desks is legislated if the employee's work requires predominantly sitting tasks. This highlights progressive thinking towards sitting and moving at work in these countries that currently isn’t widespread in the UK.

In addition, this area of research relies predominantly on intervention studies with limited qualitative research to assess employees and employers views on the concept. A recent study by Chau et al. 2019 investigated Australian perspectives on policies and practices to promote PA in the workplace from a number of different industries [23]. They sought to speak with both employees and managers on current practices and potential barriers to PA at work, but did not explicitly investigate PA in paid work time. Including not only employees but senior manager’s in such research has been shown to be important in the workplace, particularly in relation to the acceptability and feasibility of potential workplace innovations such as sit less move more strategies [24, 25]. Gaining perspectives on PA in paid work time from throughout the organisational hierarchy might be important in developing a greater understanding of the barriers to this specific concept. Therefore, the aim of this study was to explore UK employee and employer perspectives of PA in paid work time and to report the possible barriers and benefits of such an initiative.

## Methods

### Study design and recruitment

Workplaces in central Scotland with mainly desk-based employees were approached through existing contacts of the research team to take part in this qualitative study. A qualitative approach was chosen in order to gain a deeper insight into employees' perceptions, beliefs, and values on the topic of PA in paid work time and because it is suited to addressing potential cultural differences as were anticipated in the current research [26]. Focus groups with employees and middle managers were selected over other qualitative data collection methods such as surveys or interviews as they allow for a debate to occur on the topics raised and follow up questions to be asked in order to understand what were consistent or shared views and not weighted by extreme individual perspectives [27, 28]. However, for pragmatic reasons interviews were chosen with strategic managers as typically only one or two people held these positions and they were easier to arrange with their schedules.

Out of five organisations approached, three agreed to take part in the study (a University and two executive non-departmental public bodies). A local government authority and private sector organisation were unable to participate. Two one-to-one interviews were held with strategic managers, five focus groups with middle managers (*n* = 16) and nine with employees (*n* = 45) in total across all organisations. Focus groups and interviews were conducted until the research team agreed data saturation had been reached. With only two strategic managers available for interview, all managers’ views are grouped together for the purpose of this paper.

A four stage recruitment strategy was implemented: 1) Key gate keepers in the organisation (typically Human Resources Managers) were identified using an opportunist sampling approach through existing contacts of the research team and facilitated recruitment and access to the workplace; 2) Strategic managers (with an overview of the larger workplace agenda and priorities) were identified by the gatekeepers and contacted by phone and a one-to-one interview arranged; 3) Middle managers (those with a responsibility for groups of employees) were then identified by either the gatekeeper or strategic manager and invited to a focus group by email; and 4) Middle managers were then asked to send an email to their employees inviting them to take part in a focus group. All interviewees and focus group participants provided written informed consent to participate and for publication of the results. The study was approved by the Research Ethics Committee of the School of Nursing, Midwifery and Health, University of Stirling.

### Data collection

Data collection took place between May and December 2014. All interviews and focus groups were conducted separately per workplace and occurred during the working day at the participant’s place of work. Where possible, two members of the research team were present for focus groups with GR facilitating all discussions and the second member (JE, MS) taking field notes [29, 30]. For interviews, GR only facilitated and took notes. A minimum of three participants were required for a focus group to proceed with a maximum of eight employees. Focus groups and interviews lasted no longer than 60 min. Two semi-structured schedules (one for employees and one for managers) were developed by GR specifically for this study and were reviewed and edited by the research team. The schedules were developed to address key research questions relating to the acceptability, barriers, benefits and feasibility (logistical consideration) of PA in paid work time. At the beginning of the discussion participants were asked to provide some information on their job (title, type of work, PA activity in the job and other PA). Then without prompting, participants were asked to respond to an initial hypothetical statement that their workplace is providing them with the opportunity to be physically active in paid work time. They were then given a definition of what is meant by PA in paid work time for the purpose of this study; time off in addition to existing breaks to undertake PA followed by some additional parameters to help define the concept further; short (20 to 30 min), frequent (2 to 3 times per week) and including activities such as walking. Questions for both managers and employees then focused on the main research questions of acceptability, barriers, benefits and feasibility (logistical consideration) of PA in paid work time. Managers were asked these questions from a managerial perspective and as an employee themselves. Managers were also asked background questions relating to the organisation (sites, employee numbers), and current PA provision. Table 1 provides an overview of the schedules.

**Table 1:** Question schedule used in the focus group with employees and managers

**Introduction**	
Opening questions	Can you tell me job title, the type of work you do, how active your job is and any physical activity you do in your day.^a^
Initial reaction to the concept	We are investigating the hypothetical idea of providing physical activity to employees in paid working hours.^a^ What is your initial reaction to this idea? *Definition of PA in paid work time provided before continuing with questioning*
**Employee questions**	
Acceptability	Would you take part?^a^
	Could you tell me your thoughts on this if you were provided this opportunity?^a^
	Do you think it would work in your organisation?
	How supportive do you think your managers would be?
Barriers	What would stop you from taking part?^a^
	What would be the barriers to offering PA in paid time?^a^
Benefits	What would be the benefits to offering PA in paid time?^a^
	If your organisation were to invest in this idea, what do you think they would need to be shown in return?
Feasibility (logistical considerations)	What would be the logistical considerations and concerns about delivering PA in paid time?^a^
	Do you think this initiative would get the whole workplace involved?
	Would your manager take part in this and how would this affect you and your colleagues taking part?
	Should it be compulsory or voluntary?^a^
**Managers questions**	
Organisation background	How many employees and sites do you have?
	What is the current physical activity provision at the organisation?
Acceptability	(in reference to the definition of PA in paid work time) Could you tell me your thoughts on this and whether you think it would work in your organisation?
Barriers	As someone who manages others, what would be the barriers to offering PA in paid time?
Benefits	As someone who manages others, what would be the benefits to offering PA in paid time?
	If your organisation were to invest in this idea, what would they need to be shown in return?
Feasibility (logistical considerations)	What would be the logistical considerations and concerns about delivering PA in paid time?

All discussions opened with the questions in the introduction section. Employees were asked the questions in the employee section with managers asked the manager questions in addition to the employee questions marked^a^

After the discussion all participants were asked to complete a short questionnaire containing socio-demographic questions (Additional file 1). The survey was used to assess age, social economic status (Scottish Index of Multiple Deprivation - a postcode measure of material deprivation) [31], ethnic background, qualifications, employment status, hours worked, days worked, office layout, flexible working and line management responsibilities. Activity at work was measured using a validated questionnaire (Occupational Sitting and PA questionnaire) [32] and leisure time PA measured using a validated single item question [33].

### Analysis

All interviews and focus group discussions were digitally recorded and transcribed verbatim. Notes were written up immediately after the discussions. Data analysis started during the fieldwork phase of the study using the constant-comparative technique [34] so that unanticipated issues raised by the participants could be explored in subsequent interviews and focus groups. Data were analysed thematically [35]. This process involved several steps: Familiarisation with data enabled construction of a first level coding framework informed by 1) the research questions underpinning the study - acceptability, barriers, benefits and feasibility (logistical consideration) of PA in paid work time; 2) topics and issues introduced by researchers during the focus groups and; 3) topics and issues discussed by participants during interviews and focus groups. NVivo (v10) was used in the process of applying the first level coding framework to each transcript (PA). Initial coding was reviewed by GR, who identified a number of additional emergent codes or themes. Descriptive analysis and interpretation of coded data was undertaken by GR and PA through several in person discussions. During these discussions, significant overlap in themes between managers and employees were identified largely due to managers talking about themselves not only as managers but as employees. Data for managers and employees were analysed separately but reported together under the main themes and it was made clear in the text when managers' views were shared or differed from those of employees. In order to ensure validity of interpretation, a sample of coded data was selected and reviewed by other members of the research team (TG and JE). Data analysis was concluded by June 2018.

Socio-demographic data were entered into SPSS (IBM Statistics 21). Continuous data are presented as mean ± standard deviation. In text, age is also presented as median, minimum and maximum values. Categorical data are presented as *n* and %. Time spent sitting at work was calculated using the percentage of sitting time and the total hours worked.

## Results

### Participant demographics and workplace characteristics

A total of approximately 3358 employees across 46 worksites were employed at the three organisations at the time of the study although not all were invited to take part. Existing work break schedules varied both between and within organisations. One organisation had considerably better provision of onsite sports and PA facilities than the other two sites. All had sheltered bike rack spaces and links to external sports and PA providers within 2 miles of the workplace. Of the 63 participants who chose to take part in the study, 42 were female and 21 male (employee focus groups and manager focus groups/interviews both 67% female). Additional socio-demographic characteristics of employees are presented in Table 2. Only 27 participants returned the questionnaire. Of these, the median age was 43 years (23 to 58 years), with the majority living in affluent areas (80%) and University educated (81%). Most participants worked full time (81%) spending a mean of 35.9 h per week at work. Participants mostly worked in open plan offices (85%) and had no line management responsibilities (93%), with 67% describing themselves as having flexible working hours. Participants were largely in sedentary jobs and spent the most of their day sitting (87%). None of the participants reported any heavy labour at work. Leisure time PA varied greatly ranging from zero to 16 h per week, with participants achieving a mean of 3.1 h per week.

**Table 2:** Demographic and self-reported activity data of participants

Characteristics	Total
**Age** years, M ± SD (min to max)	42.9 ± 11.2 (24 to 58)
**Scottish Index of Multiple Deprivation** quintiles, (*n* = 21) *n* (%)	
1 (0–20%) most deprived	2 (10)
2 (20–40%)	3 (15)
3 (40–60%)	0 (0)
4 (60–80%)	8 (40)
5 (80–100%) least deprived	8 (40)
**Ethnic background***n* (%)	
Other	3 (11)
White Scottish	24 (89)
**Qualification***n* (%)	
University or higher	22 (81)
Certificate/diploma/trade	4 (15)
No formal qualification	1 (4)
**Employment status***n* (%)	
Full time	22 (81)
Part time	5 (19)
**Hours worked** hrs/week, M ± SD (min to max)	35.9 ± 8.3 (14 to 60)
**Days worked** days/week, M ± SD (min tomax)	4.7 ± 0.7 (2 to 5)
**Office layout***n* (%)	
Open plan	23 (85)
Own office	4 (15)
**Flexible working***n* (%)	
Yes	18 (67)
No	9 (33)
**Line management responsibilities***n* (%)	
Yes	2 (7)
No	25 (93)
**Activity at work** % of work hours	
Sitting	87
Standing	7
Walking	6
Heavy labour	0
**Time spent sitting at work** hrs/week, M ± SD (min to max)	31.4 ± 7.9 (10 to 48)
**Leisure time physical activity** hrs per week, M ± SD (min to max)	3.1 ± 3.5 (0 to 16)
Achieving > 2.5 h leisure time physical activity per week *n* (%)	14 (52)
Achieving < 2.5 h leisure time physical activity per week *n* (%)	13 (48)

Based on *n* = 27 unless otherwise stated

*NA* not applicable

### Qualitative results

To place the results in context, we first explore attitudes towards the workplace as a setting for PA in general before discussing the specific concept of PA during paid work time.

#### The workplace as a setting for physical activity

If they choose to, most employees agreed they could already be active on a work day in unpaid time such as immediately before or after work or during lunchtime. Participants’ previous or current experiences of PA in this time were discussed and it was typically ‘exercise’ type activities (i.e. swimming, exercise classes or cycling) that were undertaken. Some of these were opportunities available at onsite facilities. For these sorts of activities on a work day, participants discussed potential barriers such as logistics of needing to bring a change of clothing, time to shower and change as well as the location and provision of facilities (e.g. availability of showers).

In addition to these logistical considerations, for many, the idea of being active even if during unpaid, discretionary time but whilst at work such as during lunchtime, generated further barriers. For instance, feelings of guilt for not working were discussed:

*“I know I should go to the gym and such like, but I just feel that there’s never enough time to fit it in by the time you go down the sports centre, change, do your stuff, have a shower, change, it just completely eats over the hour and you feel that you’re…you know, somewhat taking the Michael for your hour and a half lunch, so it’s not great.” [Employee, site 3]*

This flagged up a key overarching issue regarding attitudes towards the workplace as a setting for PA and the cultural norms and attitudes towards this idea. This issue became even more pronounced when we went on to consider the concept of PA during paid work time.

#### Physical activity during paid work time

An overview of the themes relating to PA in paid worktime is presented in Table 3. Whilst questions on acceptability and feasibility were discussed, concepts raised were largely related to the main themes of benefits and barriers and are presented in this way.

**Table 3:** Overview of themes relating to PA in paid work time

**Benefits**	
Employee benefits	Improved mental health
	Improved productivity
	Improved physical benefits (e.g. fitness, energy)
	Improved perception of employer
Organisational benefits	Improved productivity
	Reduced sick leave
	Improved colleague relationships and morale
	Improved perceptions of employer
**Barriers**	
Structure and nature of the working day	High workload
	Frontline job requirements and scheduling of breaks
	Not knowing current break entitlement
	Existing flexible working arrangements
Workplace culture and norms	Resentment from colleagues
	Physical activity not accepted in the workplace
	A no break, be at your desk culture
Organisational concerns	Cost of time lost
	Public and media perceptions of spending funds

### Benefits

Both employees and managers discussed the anticipated benefits that could be gained as a result of being physically active during paid work time. These could be typically be broken into benefits for the employees and benefits for the organisation. Whilst some benefits were noted more by employees than managers (employees noted the physical benefits whilst the managers placed less emphasis on this), they were generally in agreement. Benefits noted typically included improved productivity and mental health, reducing stress, reduced sick leave and employees having more favourable perceptions of their employer. In relation to improved productivity one manager said:

*“You might well — I'd hate to promise this — but you might well get back the investment of time” [Manager, site 3]*

### Barriers

Despite widespread agreement of the anticipated benefits of PA in paid work time, the idea of actually participating in it was viewed on the whole problematic. This was for three key reasons: (i) *Structure and nature of the working day*; (ii) *Workplace culture and norms,* and; (iii) *Organisational concerns.*

(i) *Structure and nature of the working day*

*High workload*: One of the main barriers mentioned by employees was workload. Adding extra time in for PA without reduction of workload was seen as a challenge. One employee said there would be no point in taking the time for PA if a longer day is required to achieve the same work volume. Others mentioned that the main anticipated benefit they saw from this initiative, improved mental health and reducing stress, might be comprised if they were worried about their work output when away from their desk.

*“Yeah, an awful lot of people would see that yeah, this is a nice idea, but you'll still be expected to do this, this, this and this and you know “it's window-dressing” em, would be I think the criticism made, that you make this available but you don’t really, the workplace doesn't really believe, it's not going to create extra time, it's not going to ease off on the pressures on you…” [Manager, site 3]*

*Frontline job requirements and scheduling of breaks*: Having a frontline job where you are required to be present at your workstation was perceived to be a significant barrier. Often these roles are structured, with breaks having to be taken at set times. Employees in such roles noted issues such as requiring cover for their position from another member of staff and needing to have time scheduled in advance. This was seen as a significant problem in areas which are already understaffed and where resources were stretched.

“*We're really thinned down, so on a normal day, we've just got enough people, but, for example, someone calls in sick, we're at a crisis point if someone calls in sick…… I think you might get the staff would want to do it, but the fact is that there might not be people to cover if people are going to go and do exercise.” [Employee, site 3]*

*Not knowing current break entitlement:* Some employees were unsure of their official break entitlement and different departments in the same organisation had different break schedules. They discussed that such discrepancies would make it difficult to operationalise a new break allowance and it would be important to articulate what this new break would mean in practice. When discussing their current break schedule one employee mentioned having 50 min for lunch but not any other break. A colleague then responded:

*“Is it not an hour for lunch and two ten or 15 minute breaks, depending on how long you work?” [Employee, site 3]*

*Existing flexible working arrangements*: Participants mentioned that many of their colleagues work condensed hours or have arrangements in place to leave early for personal reasons like collecting children from school. These employees already work through breaks to have more time out of work. Some were also concerned that formalising PA in paid work time might result in a reduction in existing privileges relating to breaks (some of which were not formally recognised) and these existing working arrangements.

*“I mean, I do fixed hours so I work 8.30 till 4.30 because I have kids to collect, and I don’t want to be late out of the office because then just snowballs out of control otherwise, so I can manage to have one break, maybe, before lunch or whatever, but if I had another break I would be like, well that’s taking the Michael a bit, really, because I’m working those hours and I don’t really have capacity to stay on an extra hour at the end of the day, because I would feel if I’ve had another break I need to work longer, so it kind of defeats the purpose.” [Employee, site 2]*

(ii) Workplace culture and norms.

As with the idea of PA during the workday, existing workplace culture and norms would underpin whether PA during paid work time would be a feasible arrangement.

*Resentment from colleagues:* There was a perception that colleagues who feel very stressed and overworked would be particularly resentful towards other colleagues taking part in PA in paid work time. A view was expressed that if a colleague has the time to participate in PA then they must not have enough work on.

*“...you can be very, very busy, and you know, and there could be resentment when somebody is sitting there, really up to here with work. And then they see, oh that's their turn for getting off for their 20 minute exercise.” [Employee, site 1]*

*Physical activity not accepted in the workplace:* Some participants questioned whether the workplace was an appropriate setting for PA, irrespective of the type of PA e.g. walking, desk based stretching, or traditional exercise.

*“I like the idea though of the desk based ones where you can just do something. Though having said that I do remember a time when I was sitting doing my neck rolls because I was getting quite stressed and the person opposite he goes, what on earth are you doing? So that just totally broke my thing.” [Employee, site 3]*

*” A business is a business at the end of the day… that was what I would argue, there are limits to what you can support” [Employee, site 1]*

*A no break, be at your desk culture*: Employees discussed the idea of a ‘look busy’ culture. There was a feeling that employees at their desks are working hard and those who appear to be in for long days and not taking breaks are working the hardest. There was a concern that people who took up the opportunity to be active in paid work time would be perceived as not hard working.

*“It’s more if there’s an expectation of you to be at your desk and if somebody comes and needs some information urgently which is the kind of responsive mode we’re in, I wouldn’t want anybody to say to those people, she’s off on her exercise break. That would be a barrier to me taking an exercise break.” [Employee, site 3]*

People also reported a ‘no break’ culture and not making use of existing break entitlements. Merely providing these people with more time even if specifically for PA is not likely to make any difference to their behaviours.

*“I feel like this is, speaking for myself, this (PA in paid work time) would be like a secondary step. The first step is to be really proactive in getting people to use their lunchtime properly.”[Employee, site 3]*

Employees expressed feeling guilty about taking existing breaks they were entitled to and even more guilt if this time were to be used for PA. The idea of a break for PA in addition to their existing break time generated even more discussions of guilt and being away from the desk at unexpected times.

*“It would be nice to be able to fit it (PA) into your work day without feeling guilty that you’re not at your desk.” [Employee, site 3]*

However, quite often when people talked about the guilt, this fear was internalised. Even those with colleagues and managers who were perceived as potentially supportive expressed some ambivalence. When asked who they thought would be disapproving one employee responded:

*“No-one, I think it’s your inner voice really, isn’t it? You’ve just got to work...” [Employee, site 3]*

(iii) *Organisational concerns*

*Cost of time lost:* Managers more often that employees reported financial cost to the organisation from potential time lost to additional breaks as a significant barrier. It was often described in terms of man hours or ‘full time equivalents’ that would be lost.

*“I mean if you add 30 times 1,200 people it's quite a lot of time, you know, every day, or twice a week; you know if you do the maths it looks like a lot of time” [Manager, site 1]*

*Public and media perceptions of spending funds:* Executive non-departmental public bodies at both a managerial and employee level had concerns over public and media perception of spending public resources on employee PA (even when the potential benefits and financial cost savings to the organisation were acknowledged). This was not a consistent theme raised by those at the University.

“*Even if we do change the culture and do 15 minutes of exercise, it’s the public perception, as well as an organisation, you are there to provide a service … we are paid by public funds and we need to be seen to be working.” [Employee, site 2]*

## Discussion

The aim of this study was to explore UK employee and employer perspectives of PA in paid work time and to report the possible barriers and benefits of such an initiative. The underlying rationale was that this might overcome one of the main barriers to PA: perceived lack of time. This study found that there were many anticipated benefits from such an initiative, including productivity and reduced sick leave. Despite this, several additional barriers were raised including the structure and nature of the working day and workplace culture and norms that would need to be addressed before such an initiative would work in practice.

Whilst it was anticipated that managers might have reservations about the concept in relation to perceived ‘lost work’, it was though that employees themselves would be largely supportive for the opportunity to have a break from work while enjoying the benefits of PA. However, what was not anticipated was the strength of the cultural and attitudinal factors which mitigate against the concept and that both employees and managers would share similar perceptions on this. These comments underlined the importance of taking into account existing ingrained workplace cultures not only relating to PA but around the working day in general.

Other studies looking at reducing sedentary time at work have reported similar findings [36, 37]. In a group of 20 sedentary office workers in Australia, Hadgraft et al. (2016) reported workload and organisational social norms as key barriers to employees aspiring to sit less and move more at work. Unlike the current study, they reported a workplace culture that did support the use of existing breaks or moving around the office if it was for a permissible reason such as getting a coffee or for a work task. However, if you were moving around the office for something other than work or if you were seen taking part in exercise related activities in the office such as stretching, similar feelings of being self-conscious as found in the present study were reported. Changing attitudes in the workplace towards being away from the desk and assisting employees to take breaks they are entitled to would be the first steps required prior to implementing PA in paid work time.

When discussing potential barriers of PA in paid work time for employees, high workload was noted as the most significant barrier across all workplaces and employees. Bale et al. (2015) conducted a quantitative analysis of environmental barriers to 30 min of daily exercise provided in paid work time [38]. They found that those who reported having too much work were three times less likely to use their PA time than others who reported having more manageable workloads. Managers in the present study also noted a similar concern with regards to the potential cost of lost time with employees potentially working less time each day. Both employees and employers did acknowledge that there was an anticipated potential benefit to productivity which could counteract this lost time or workload. These results suggest that merely providing additional time for PA may without adjusting employees’ workload or demonstrating a positive effect on productivity would not be enough to allow employees to take up this opportunity or the workplace to buy into the concept.

The results of the current study also suggest other aspects relating to the structure and nature of the working day may need to be considered when providing time for PA during paid work time. Participants who described themselves as having more frontline responsibilities suggested they were restricted with when they could use this time and reliant upon both their managers scheduling, and their colleagues providing, cover. In contrast, those with more autonomy in their jobs suggested the need to ensure activity time is protected and not taken over by work, with motivation potentially a more relevant barrier for this group. Employees’ personal circumstances also vary greatly even within the same job which may influence their ability to use time at work for PA. Someone who condenses their time for caring responsibilities for example may need to be assured these arrangements wouldn’t be changed. However, these factors are largely related to logistical considerations and could be seen as not only easier to address but secondary to changing workload and workplace culture.

Support for this idea throughout the organisational hierarchy is essential and the idea of PA in paid work time being ‘permissible’ is clearly important to implementation. This is especially relevant given concerns raised about what colleagues and managers might think of this idea. However, engaging workplaces and strategic managers to take part in this qualitative study was difficult, with only two strategic managers interviewed. Even those managers who did take part expressed the potential need to see benefits as a result of taking time out of paid work for PA. Combined, the results might suggest that the workplace is not currently an ideal setting for PA with significant cultural shifts required in order for such an initiative to work.

This study has several limitations which need to be considered. Although the study gained the perspectives of PA in paid work time from both active and inactive employees, the sample were relatively homogenous - largely affluent, not ethnically diverse, highly educated and from only three different organisations. More research is needed to assess whether the barriers presented in the current study are the same in a wider range of workplaces (private sector, small to medium enterprises) and in a more diverse employee population. This is critical given that those who are less educated or in lower paid jobs may have different views on their job autonomy and ability to control how their time at work is allocated. Whilst the current study investigated the views of predominately desk-based employees, many workplaces have both sedentary and active occupations. It is important to gain the perspectives of those in occupations where employees are on their feet or engaging in labour as part of their work in addition to those in sedentary roles. This is for two reasons. Firstly, although those in sedentary occupations are likely to gain significant benefits of PA in paid work time, providing time off to one group and not others is unlikely to be acceptable in workplaces. Indeed, participants in the current study highlighted the need for clear operationalisation of these concepts. Secondly, given recent systematic review evidence that suggest that physically demanding jobs such as labourers may have an increased risk of all-cause mortality (although more for males than females), PA in paid work time might also be beneficial in active occupations [39].

Future research should also address ways to overcome the barriers presented in the current study and look to develop strategies and interventions that aim to implement PA in paid work time. One such approach could be looking at even shorter periods of time, such a five-minute breaks, and the types of activity that might be feasible and acceptable during that time. Further qualitative research on how to change attitudes towards PA at work is required to fully understand how this can be achieved. Researchers should also look to work more closely with workplaces to co-create potential interventions and to report case-studies and process evaluations to first detail *how* PA in paid work time could be implemented more than *if* it can be effective at changing outcomes.

## Conclusion

This study suggests that even if PA opportunities were provided to employees in paid work time, significant barriers for both employees and employers would need to be addressed in order for such as initiative to be successful. Whilst some barriers, such as the logistics of providing cover for front line staff, can potentially be overcome, there is an urgent need to challenge current attitudes and culture towards PA at work. Until this occurs, PA in paid work time in unlikely to be successful with workplace culture a key point for consideration in any workplace PA interventions whether in paid work time or not.

## Supplementary information

**Additional file 1.** Short questionnaire, Socio-demographic, work environment and physical activity questions.

## Data Availability

The datasets used and/or analysed during the current study are available from the corresponding author on reasonable request.

## Abbreviations

PA: Physical activity
NA: Not applicable
