# Supplementary material for: Physical activity in paid work time for desk-based employees: a qualitative study of employers’ and employees’ perspectives
Source: BMC Public Health. 2020 Apr 6;20:460. doi: 10.1186/s12889-020-08580-1 (PMC7137494; doi:10.1186/s12889-020-08580-1)
Supplement: Supplementary file 1 — Additional file 1. Short questionnaire, Socio-demographic, work environment and physical activity questions. [file 12889_2020_8580_MOESM1_ESM.pdf]

MRC Medical Research Council

UNIVERSITY OF STIRLING

Incentivised Physical Activity in Workplaces (IPAWs) Survey

Tell us about you

In this section we want to find out a bit about who you are.

1. **In what year were you born?** [ ]

2. **What is your home postcode?** [ ]

3. **What is your gender?**
   Please choose one of the following:

- [ ] Male
- [ ] Female
- [ ] Other. Please specify: ______________________________

4. **How would you describe your cultural or ethnic background?**
   Please choose one of the following:

- [ ] White Scottish
- [ ] Other white British. Please specify: ______________________________
- [ ] Other. Please specify: ______________________________

5. **What is the highest qualification that you have ever COMPLETED?**
   Please choose one of the following:

- [ ] No formal qualification after school
- [ ] Trade, technical certificate, apprenticeship or diploma
- [ ] University degree or higher

Tell us about your job

In this section we want to find out a bit about your work and job.

6. **Which of the following best describes your current employment situation?**
   Please choose one of the following:

☐	Employed full time
☐	Employed part time
☐	Zero hours contract
☐	Other. Please specify: ____________________

7. **How many hours did you work in the last 7 days?**

   ____________ **HRS**

8. **During the last 7 days, how many days were you at work?**

   __________ **DAYS**

9. **How would you describe your typical work day in the last 7 days?**
   This involves only your work day, and **DOES NOT INCLUDE** travel to and from work, or what you did in your leisure time.
   Please indicate the percentage of time in each of the following:

**A.**	**Sitting (including driving – but not too and from work)**	__________	**%**
**B.**	**Standing**	__________	**%**
**C.**	**Walking**	__________	**%**
**D.**	**Heavy labor or physically demanding tasks**	__________	**%**
	These should total	***100%***	

10. In the past 7 days, how much time have you spent doing physical activity which was enough to raise your breathing rate?

This may include sport, exercise and brisk walking or cycling for recreation or to get to and from places, but **DOES NOT INCLUDE** housework or activity as part of your job.

___________ **HRS**

11. What is your office layout?

Please choose one of the following:

- ☐ Open plan
- ☐ Own office
- ☐ Other. Please specify: ______________________________

12. Would you describe yourself as having flexible working hours?

Please choose one of the following:

- ☐ Yes
- ☐ No

13. Do you have any line management responsibilities?

Please choose one of the following:

- ☐ Yes
- ☐ No

This is the end of the survey, thank you for participating!
